# 2123. Novel Antimicrobial Polycarbonate Coating for Intravenous Connectors

**DOI:** 10.1093/ofid/ofad500.1746

**Published:** 2023-11-27

**Authors:** Y Lan Truong, Bahgat Z Gerges, Joel Rosenblatt, Issam I Raad

**Affiliations:** UT MD Anderson Cancer Center, Houston, Texas; MD Anderson UT, Missouri City, Texas; MD Anderson UT, Missouri City, Texas; MD Anderson UT, Missouri City, Texas

## Abstract

**Background:**

IV connectors are ubiquitous access junctions in vascular access circuits. Unlike vascular catheters and IV lines made from soft polymers, connectors are made from rigid polycarbonate (PC) to prevent deformation during manipulation. In accessing PC connectors (PCCs) for infusion, PCCs are vulnerable to becoming microbially contaminated. Here we report development of a novel antimicrobial polycarbonate coating for preventing microbial colonization of IV connectors.

**Methods:**

The FDA recently approved a new antimicrobial polyurethane catheter coating that comprised Minocycline (M), Rifampin (R), and Chlorhexidine (C). We spray-coated MRC onto PCCs as sequential laminate layers of M, R and C in PC or blend PC and polycarbonate-urethane copolymers. MRC coated PCCs were immersed in tubes filled with 50% serum + 50% saline to simulate elution of the coatings. After 7 days (typical interval of use prior to replacement of IV connectors), eluted connectors were exposed to challenge inocula of key CLABSI pathogen clinical isolates for 24 hours and the number of colonizing microbes were enumerated by sonication, serial dilution, plating and colony counting. Non-coated PCCs were positive controls.

**Results:**

Table 1 presents number of viable colonies recovered after 7 days elution and following 24 hour microbial challenges with Methicillin-resistant *Staphylococcus aureus* (MRSA), *Pseudomonas aeruginosa* (PA) and *Candida albicans* (CA).

**Figure:**
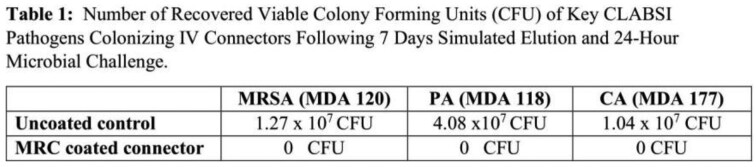

**Conclusion:**

The absence of microbial colonization of the MRC IV connectors demonstrates the efficacy and durability of the novel MRC polycarbonate laminate coating. MRC coated connectors merit further testing in vitro and in vivo for their potential to prevent CLABSI.

**Disclosures:**

**Joel Rosenblatt, PhD**, Novel Anti-Infective Technologies, LLC: Licensed Technology **Issam I. Raad, Distinguished Professor**, Novel Anti-Infective Technologies, LLC: Technology License

